# Depot Typical Antipsychotics versus Oral Atypical Antipsychotics in Relapse Rate Among Patients with Schizophrenia: A Five -Year Historical Cohort Study

**Published:** 2014

**Authors:** Hamid-Reza Ahmadkhaniha, Shahab Bani-Hashem, Masoud Ahmadzad-Asl

**Affiliations:** 1 Assistant Professor, Department of Psychiatry, Mental Health Research Center, Tehran Psychiatry Institute, Tehran University of Medical Sciences and Health Services, Tehran, Iran.; 2 Psychiatrist, Department of Psychiatry, School of Medicine, Kerman University of Medical Sciences and Health Services, Kerman, Iran.; 3 Psychiatrist, Department of Psychiatry, Mental Health Research Center, Tehran Psychiatry Institute, Tehran University of Medical Sciences and Health Services, Tehran, Iran

**Keywords:** Antipsychotic Agents, Dopamine Receptor Antagonists, Long-Acting Drugs, Schizophrenia, Serotonin Dopamine Antagonists

## Abstract

**Objective:** The present study aimed to review the relapse rate in patients with schizophrenia treated with orally taken atypical agents (serotonin dopamine antagonists, SDAs) and depot preparation of conventional (typical) antipsychotics.

**Methods:** In this historical cohort study, mean relapse per month (MRM) index, duration between initiation of antipsychotic treatment and the first relapse episode, and the time gap between successive relapses were compared between 84 patients on SDAs-except clozapine (group 1) and 81 others on depot typical antipsychotics (group 2).

**Results: **The two groups were comparable regarding mean (±SD) MRM index [0.033 (±0.004) in group1 and 0.044 (±0.05) in group 2; p = 0.345]. Mean (±SD) duration of time between initiation of maintenance treatment and the first relapse was 15.5 (±13.67) months in group 1 and 16.40 (±15.31) months in group 2, (p = 0.876). Mean (±SD) duration of remission periods between successive relapses were 17.92 (±14.2) and 15.8 (±16.9) months for group 1 and group 2, respectively (Mann-Whitney test, (p = 0.048).

**Conclusion:** Orally taken atypical antipsychotics were able to keep the duration of remission periods between successive relapses more prolonged compared to depot conventional preparations. This could be added to their other remarkable benefits especially if the patient is expected to experience multiple relapses.

**Declaration of interest:** None.

## Introduction

Schizophrenia is a heterogeneous disorder that needs specific treatment strategy. Bringing the acute phase of schizophrenia into the remission as soon as possible and preventing the relapse is essential to maintain functional state and well-being for patients. Several factors are known to influence the acute course of schizophrenia and relapse including non-modifiable factors such as gender and age on onset of symptoms and those that could be modified to attain more effective and efficient management plans (1, 2). Antipsychotic maintenance treatment in addition to some non-pharmacologic interventions such as psycho-education, telecommunications, family education, using information technology services, etc. play a key role in achieving the treatment goals (3-5).

The patients' adherence to their antipsychotic medications is the best known predictor for decreasing the chance of relapse; however, it still is the most challenging issue in long-term management of schizophrenia that is directly linked to impaired long-term outcomes and cost of the disease (6-8). 

Choosing an effective, safe and well-tolerated antipsychotic agent is sometimes complicated due to variety of available drugs and specific considerations for every individual patient (9). Second generation (serotonin dopamine antagonists; SDA) or atypical antipsychotic agents, due to their proven better control on positive and negative symptoms, less incidence of extra-pyramidal adverse effects and fewer relapses, are considered the treatment of choice in schizophrenia (10); although their clinical advantage is sometimes limited due to issue of adherence (11-14). Long acting depot antipsychotics are not only able to reduce the differences in peak and through plasma levels of the drug, but also provide a more reliable rout of drug delivery. The more favorable results of maintenance therapy with these injectable antipsychotics are mainly due to an improved patient’s adherence (-). Atypical long-acting agents are to be considered a favorite option in maintenance therapy, revealing both the benefits of oral atypical antipsychotics, in terms of symptom control and fewer side effects, and depot agents, in terms of better adherence (11, 19, 20). 

Since atypical long-acting agents are not available widely, for example in low and middle income countries, it is important to have evidence that help to choose between "limited efficacy and treatment-limiting side effects of conventional depot neuroleptics" and "less improved adherence of atypical antipsychotics". The present study aimed to compare these medications in patients with schizophrenia.

## Materials and Methods

This study was designed and done in Iran Psychiatry Hospital, Tehran, Iran as a referral psychiatric mental health and inpatient service in Tehran. In this historical cohort study, medical records of 1,262 patients were reviewed with the diagnosis of schizophrenia, confirmed during at least one hospitalization in an educational hospital between 2001 and 2006. Known cases before 2001, patients who dropped follow up in less than six months from making the diagnosis, patients for whom the diagnosis was changed from schizophrenia to other psychiatric disorders(such as schizoaffective disorder, substance-induced psychosis, delusional disorder, schizophreniform, and major depression disorders), and those who received combination therapy (e.g. simultaneous SDA and dopamine antagonists) were excluded. Finally, 165 patients remained to be studied; of whom 81 were treated with orally taken atypical antipsychotics, and 84 with depot typical antipsychotics.

Rehospitalization and/or evident relapse of symptoms in a previously symptom-free patient, recorded in outpatient follow up notes, were considered as the main indicators of relapse. The relapse was defined as presence of at least two out of five characteristic symptoms of criterion A in *Diagnostic and Statistical Manual of Mental Disorders*, 4^th^ ed. (DSM-IV) Text revision (21). Patients, who dropped out treatment at anytime, for at least one month, were considered as non-adherent. 

To reduce the confounding effect of length of fallow up period on frequency of relapse episodes, we defined a Mean Relapse per Month (MRM) index $\left(\frac{\mathrm{number of relapses}}{\mathrm{follow up Time}(\mathrm{month})}\right)$ . Duration between initiation of antipsychotic treatment and the first relapse episode, the time gap between successive relapses, in addition to age, gender, marital state (at the time of the diagnosis), family history, and substance abuse were compared between the two groups.

MRM index, duration between initiation of antipsychotic treatment and the first relapse episode, and the time gap between successive relapses were compared between 84 patients on oral SDAs-except clozapine (group1) and 81 others on depot typical antipsychotics (group 2).

The chi-squares test was used to compare the proportion of variables between the study groups. Mann-Whitney U and Kruskal-Wallis tests were performed for comparison of means. 

The "Research Ethics Committee" of Research Center for Mental Health, Iran University of Medical Sciences approved the study according to ethical considerations.

## Results

The socio-demographic characteristics of 165 enrolled patients by the type of maintenance therapy are summarized in table 1. There was no statistical significant difference in study groups regarding their age, sex, family history, marital status, and substance abuse.

The frequency of non-adherence was 68 (41.2%) in all enrolled patients. About 33(20%) of the patients in group 1 and 35(21.2%) of the patients in group 2 dropped out the maintenance therapy for at least one month after controlling acute course of the disease (χ^2^, p = 0.904).

About 92 (55.8%) of the patients, 43 (26.1%) on SDAs and 49 (29.7%) on conventional depot, experienced at least one relapse episode (min = 0, max = 6) (χ^2^, p = 0.498). 

The calculated mean (±SD) for MRM index was 0.03 (±0.049) in all the enrolled patients, 0.033 (±0.004) ingroup1 and 0.044 (±0.05) in group 2 (Mann-Whitney; p = 0.345).

The earliest relapse evolved one month after acute course; both in SDA and Depot groups. The longest duration of remission was 72 months; 54 and 72 months in SDA and depot groups, respectively.

Mean (±SD) duration of time between initiation of maintenance treatment and the first relapse was 15.78 (±14.4) months in total study population, 15.5 (±13.67) months in group 1 and 16.40 (±15.31) months in group 2 (Mann-Whitney, p = 0.876). 

Mean (±SD) duration of remission periods between successive relapses was 16.8 (±15.72) months. Mean (±SD) of remission period attained by SDAs and depot agents were 17.92 (±14.2) and 15.8 (±16.9) months, respectively (Mann-Whitney, p = 0.048).

**Figure 1:**
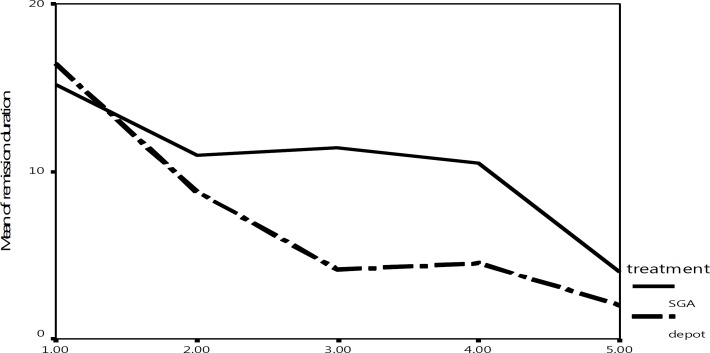
Mean duration of remission between successive relapses in atients with schizophrenia treated with SDA (Serotonin dopamine antagonists) and depot typical antipsychotics

Mean duration of remission in patients with different relapse episodes (from one to five episodes) for groups 1 and 2 declined from 15.15 and 16.4 months respectively at the first episode to 4.0 and 2.0 months respectively at the fifth episode and there was no statistical significant different between treatments group with respect to mean duration of remission in patients with different episode numbers (Figure 1).

**Table 1:** Socio-demographic characteristics of 165 enrolled patients with schizophrenia treated with SDA and depot typical antipsychotics

	**SDA** † **(n = 81)**	**Depot DA** ‡ **(n = 84)**	**Total** **(n = 165)**	**P-value**
**Age: Mean (±SD)**	27.59 (±1.05)	30.29 (±0.92)	28.96 (±9.08)	0.057
**Gender**				
**Male, number (%)**	66 (81.5%)	68 (80.9%)	134 (81.2%)	0.931
**Female, number (%)**	15 (18.5%)	16 (19.1%)	31 (18.8%)	
**Family history**				
**Yes, number (%)**	34 (42.0%)	36 (42.9%)	70 (42.4%)	0.909
**No, number (%)**	47 (58.0%)	48 (57.1%)	95 (57.6%)	
**Marital state**				
**Single/divorced, number (%)**	66 (81.5%)	65 (77.4%)	131 (79.4%)	0.515
**Married, number (%)**	15 (18.5%)	19 (22.6%)	34 (20.6%)	
**Substance abuse**				0.853
**Yes, number (%)**	24 (29.6%)	26 (30.9%)	50 (30.3%)	
**No, number (%)**	57 (70.4%)	58 (69.1%)	115 (69.7%)	

† Serotonin dopamine antagonists, atypical antipsychotic agents;

‡ Dopamine antagonists, typical antipsychotics

## Discussion

According to our findings, administration of oral atypical antipsychotics and injectable depot preparations of conventional antipsychotics were comparable regarding "adherence to treatment","MRM index", and "duration of time between starting the maintenance treatment and first relapse episode".

Although the present study had an observational design and there was no treatment randomization, the statistical analysis revealed that both groups were comparable in socio-demographic factors that could influence the results; therefore the differences between the groups could mainly be imputed to the type of treatment.

The vast majority of current studies support using long-acting atypical antipsychotics compared to oral SDAs or typical agents (22-24). There are few studies aimed on comparison between SDAs and depot conventional antipsychotics, but in areas that long-acting atypical agents may not be accessible easily, the decision to choose between SDAs and depot typical agents still matters. Conley et al. compared the risk of rehospitalization in patients on clozapine, risperidone, and olanzapine with those on fluphenazine decanoate and haloperidol decanoate. According to their findings, 1-year risk of readmission is comparable in patients on SDAs to those treated with fluphenazine decanoate, but lower than patients on haloperidol decanoate (25). Some studies suggest that depot antipsychotics compared to orally taken antipsychotics show relatively better outcomes in reducing relapse rate (17, 26). While the findings of a clinical trial conducted by Gaeble et al. revealed no difference between SDAs (risperidone) and haloperidol in relapse prevention (27).

In spite of all controversies (28), one commonly accepted principle is that patients should receive antipsychotic maintenance therapy (9). We suggest oral atypical antipsychotics for this purpose. These agents are shown to be able to keep the duration of remission periods between successive relapses more prolonged compared to depot conventional preparations. This could be added to their other remarkable benefits especially if the patient is expected to experience multiple relapses.

Our findings shed more light in complexity of treatment of schizophrenia and running further clinical and community based investigations regarding efficiency of different therapeutic medications would help clinicians in more evidence based management of patients with schizophrenia.

Due to complexity of the disease, analysis of just one factor such as relapse or remission is not sufficient to make the right treatment choice. Many factors such as the length of acute course of the disease, medications prescribed in previous courses, individual pre morbid conditions and socio-demographic status influence the prognosis of patients and should be considered when choosing the treatment (29, 30). But in a retrospective study that psychometric criteria such as "socio-occupational functioning assessment" or "Positive and Negative Syndrome Scale for Schizophrenia" are not easy to assess remission and relapse are of most reliable indicators of treatment efficiency.

This study had some limitations. Considering its retrospective and record-based design, we were not able to check the reason for depot typical or oral atypical antipsychotics prescription for each patient and this could potentially confound findings. Depot antipsychotics maybe associated with non-adherence with oral antipsychotics that could affect results (31). This is because that one can speculate those patients with initial non-adherence to oral antipsychotics were prescribed with depot drugs and we could not deal with this in study design and analysis. We also could not address the reason for drop-out, medications side effects and tolerability in our patients and these also potentially could be a source of bias. 
